# Benefits of Group Living Include Increased Feeding Efficiency and Lower Mass Loss during Desiccation in the Social and Inbreeding Spider *Stegodyphus dumicola*

**DOI:** 10.3389/fphys.2016.00018

**Published:** 2016-02-02

**Authors:** Bram Vanthournout, Michelle Greve, Anne Bruun, Jesper Bechsgaard, Johannes Overgaard, Trine Bilde

**Affiliations:** ^1^Department of Bioscience, Aarhus UniversityAarhus, Denmark; ^2^Department of Plant Science, University of PretoriaHatfield, South Africa

**Keywords:** sociality, spider, group living, ecophysiology, temperature dependent effects

## Abstract

Group living carries a price: it inherently entails increased competition for resources and reproduction, and may also be associated with mating among relatives, which carries costs of inbreeding. Nonetheless, group living and sociality is found in many animals, and understanding the direct and indirect benefits of cooperation that override the inherent costs remains a challenge in evolutionary ecology. Individuals in groups may benefit from more efficient management of energy or water reserves, for example in the form of reduced water or heat loss from groups of animals huddling, or through reduced energy demands afforded by shared participation in tasks. We investigated the putative benefits of group living in the permanently social spider *Stegodyphus dumicola* by comparing the effect of group size on standard metabolic rate, lipid/protein content as a body condition measure, feeding efficiency, per capita web investment, and weight/water loss and survival during desiccation. Because energetic expenditure is temperature sensitive, some assays were performed under varying temperature conditions. We found that feeding efficiency increased with group size, and the rate of weight loss was higher in solitary individuals than in animals in groups of various sizes during desiccation. Interestingly, this was not translated into differences in survival or in standard metabolic rate. We did not detect any group size effects for other parameters, and group size effects did not co-vary with experimental temperature in a predictive manner. Both feeding efficiency and mass loss during desiccation are relevant ecological factors as the former results in lowered predator exposure time, and the latter benefits social spiders which occupy arid, hot environments.

## Introduction

Elucidating the underlying factors that give rise to group living remains a challenging task because many selective forces could potentially affect the cost and benefits. Moreover, the maintenance of group living depends critically on these net benefits outweighing the costs that an individual experiences from being in a group. While group living is associated with a number of costs such as increased competition for food resources (Elgar, 1986; Grand and Dill, 1999), intensified conflict over reproduction (Huchard and Cowlishaw, 2011), and, in some cases, deleterious effects of inbreeding (Charlesworth and Charlesworth, 1987; Charlesworth and Willis, 2009), these must be outweighed, from an evolutionary perspective, by benefits such as reduced predation risk (Hamilton, 1971; Sorato et al., 2012; Unglaub et al., 2013) and increased foraging success (Ward and Zahavi, 1973; Stander, 1992). In addition, there is evidence that cooperation can increase fitness by lowering energy and/or resource requirements for certain tasks (Muradian et al., 1999; Tojo et al., 2005), although such energetic benefits of group living are arguably less well-explored, particularly for ectotherms.

The energetic consequences of group living may depend on the environmental conditions experienced by the organisms. For example, in endotherms, group living behavior such as huddling allows animals to conserve heat (Gilbert et al., 2010), thereby providing a distinct energetic advantage, especially in cold environments. In contrast, while huddling behavior is also displayed in tropical endotherms, it does not always provide a distinct energetic advantage in these environments, which already have an ample supply of heat energy. However, it is likely that the beneficial effects of these behaviors are strongly dependent on ambient temperature experienced by the group. As ectotherms do not expend energy to maintain a constant body temperature huddling has been suggested to provide benefits in the form of a reduced rate of water loss (Broly et al., 2014) and a reduced metabolic rate (Tojo et al., 2005).

The benefits and costly aspects of group living in some taxa, such as spiders, remain underinvestigated, especially on the level of energetic effects of group living. Social spiders occupy a shared nest and capture web, and they cooperate in prey capture and brood care (Aviles, 1997; Lubin and Bilde, 2007; Aviles and Purcell, 2012). They are further characterized by an inbreeding mating system and meta-population dynamics caused by frequent local extinctions and colonizations, factors that result in high levels of homozygosity, genetic homogenization and reduced genetic diversity, both at the population and species level (Agnarsson et al., 2013; Settepani et al., 2014; Settepani, 2015). These population genetic characteristics may result in deleterious genetic effects (Charlesworth and Charlesworth, 1987; Charlesworth and Willis, 2009) and ultimately a reduction in the ability to adapt to environmental change or pathogens (Bijlsma and Loeschcke, 2012) representing one of the major costs of group living in spiders.

In contrast, a number of benefits have been associated with group living in social spiders (Lubin and Bilde, 2007, and references herein). Social spiders cooperate in building the nest, which functions as a protective retreat against predators and a buffer against environmental fluctuations (Henschel et al., 1992; Henschel, 1998), and survival increases with increasing nest and group size (Bilde et al., 2007). Moreover, spiders share web maintenance and web cleaning by removal of debris. Group living may also provide a foraging benefit by allowing spiders to capture larger prey items, which they feed on cooperatively through extra-oral feeding (Yip et al., 2008; Majer et al., 2013; Majer, 2015).

However, the energetic benefits and costs of group living in social spiders have not yet been explored.

In this study, we use individuals and groups of the African social spider *Stegodyphus dumicola*, kept at different temperatures, to explore the consequences of group living for a range of bioenergetics variables. Social spiders within the genus *Stegodyphus* are some of only a handful of spider species that exhibit permanent group living and cooperative behavior (Lubin and Bilde, 2007). We assessed benefits of group living based on a number of performance measures.

- A straightforward measure of energy requirement is the standard metabolic rate (SMR), here defined as the maintenance metabolism of a fasting and resting ectotherm at a certain temperature and therefore indicating the minimal requirements for sustaining cellular processes (Brett and Groves, 1979). We investigate whether individuals in groups show a relatively lower SMR compared to solitary individuals implying reduced energy demands, which may be beneficial if it reflects relaxed costs without loss of fitness. However, because SMR represents the summed costs of all biological activities it may be difficult to interpret this in a clear cost/benefit setting. Indeed, studies of SMR in relation to group size in arthropods have reported unchanged, reduced, or elevated SMR, rendering this question unresolved (Anderson, 1993; Tojo et al., 2005; Schoombie et al., 2013).
- Closely linked to energy requirement is the energy reserve an individual spider has in terms of lipid and protein content, which gives an indication of starvation endurance (Wilder, 2011). If these reserves are more efficiently managed, leading to a lower consumption of lipid and proteins in grouped vs. solitary individuals, this would imply a reduced energetic cost.
- One example of a relaxed cost of group living could be the reduced production of digestive enzymes (Schneider and Bilde, 2008). Although group feeding can be costly from the viewpoint of an individual due to higher competition, increased extraction efficiency can be an important benefit. As spiders have extra-oral digestion, and inject digestive enzymes into the prey, the amount of enzymes from multiple individuals could be more efficient and hence lead to a lower enzyme production per spider. Additionally, more efficient feeding in groups could have other benefits such as reduced exposure to predators. For this reason, we investigated if feeding is more efficient in grouped vs. solitary spiders.
- As the production of silk is a costly, yet key aspect of spider ecology (Tanaka, 1989), reducing the amount of silk in groups can be a clear energetic advantage (Majer, 2015). We therefore investigated the web building investment for solitary and grouped spiders.
- Social spiders usually occupy the space within a nest structure at high densities. In arthropods, aggregation behavior is often seen to lower susceptibility to dehydration as it reduces the body surface area exposed to the air (Allee, 1926; Broly et al., 2013) and can create a local microclimate of increased humidity for all group individuals, thus decreasing the water loss rate (Schliebe, 1988; Yoder et al., 2002). This has been verified in a variety of organisms where positive effects of group size were found in terms of reduced weight and water loss and increased survival (Glass et al., 1998; Ivarsson and Jonsson, 2004; Rojas et al., 2013). This can be particularly relevant for several social spider species (including *S. dumicola*) as they inhabit periodically arid areas. We therefore measured water loss rate and desiccation resistance in different-sized spider groups, asking whether spiders in larger groups lose less water and show increased survival and thereby resistance to desiccation compared with single individuals.

## Materials and methods

### Study organism

*Stegodyphus dumicola* is a social spider species with a wide distribution range across southern Africa (Majer et al., 2013). They build nests, often in trees, in which the spiders seek shelter and live. While the nests are probably effective at defending spiders from predators (Henschel, 1998), little is known of their role in sheltering from environmental extremes. Temperatures varying between below freezing and above 55°C have been recorded inside the nests (Soydaner, 2013). Humidity levels in the nests also vary, with values between 0 and 100% RH having been recorded (Soydaner, 2013), suggesting that spiders experience a large range of environmental conditions.

### Spider rearing and experimental design

*Stegodyphus dumicola* colonies were collected from five populations in South Africa (Supplementary Table S1). Several colonies were collected per population and transported to Denmark by air and kept in a laboratory at Aarhus University in transparent plastic containers (11 × 17 × 17 cm) with mesh-covered lids that allowed airflow. The spiders were fed to satiation three times a week with houseflies (*Musca domestica*) and crickets (*Gryllus bimaculatus*) and their webs wettened with water mist twice a week. Due to logistical constraints, different populations were used for different sets of experiments, and these populations differed slightly in developmental status, although most populations showed overlap in body size and thereby in developmental stage (Supplementary Table S1). Only female spiders were used for experiments. It is unlikely that our results were biased by differences in population origin as there is very little genetic differentiation between populations of *S. dumicola* (Settepani, 2015).

We examined the effect of group living on i) SMR, ii) lipid/protein content (starvation tolerance and energy balance) iii) feeding efficiency, iv) web building efficiency, and v) desiccation tolerance and water balance. For all experiments, we used a blocked design that corrected for colony effects. Details of the five experimental setups can be found in the Supplementary Table S2.

### Standard metabolic rate

Two experiments were performed to assess the effect of group size on SMR while simultaneously investigating experimental and acclimation temperature effects (see below). The first experiment compared the SMR of single individuals to those of groups of five spiders, while the second experiment also included groups of 20 spiders. In the first experiment we tested whether group size had an effect on SMR, and whether the effect of group size on SMR was dependent on thermal acclimation. Therefore, we used an experimental design where spiders were first acclimated to either 22 or 30°C for 12 days. After this, spiders from both acclimation groups were randomly assigned to measurements of SMR at either 22 or 30°C. Using this fully factorial design we had two group sizes (one or five individuals), two acclimation temperatures (22 or 30°C) and two measurement temperatures (22 or 30°C) resulting in eight different treatment groups.

Prior to onset of the first experiment, spider colonies were fed to satiation and individuals from nine colonies were randomly divided over the eight treatment groups (acclimation temperature × SMR temperature × group size), resulting in nine replicates per treatment combination. Spiders were weighed to the nearest 0.01 mg using a Sartorius Laboratory Balance (type 1712; Göttingen, Germany) and placed in cylindrical metabolic glass chambers covered with a mesh at both ends. Spiders remained in these temperature chambers for 12 days without food, but were sprayed with water every 2nd day. After 12 days, metabolic rate was measured from the rate of CO_2_ production (V_*CO*2_) over a total experimental period of 6 days. Twelve groups were measured daily (six replicates with five and six replicates with one spider). Standard metabolic rate (SMR) was estimated from the rate of CO_2_ production using intermittent closed respirometry. The experimental setup was similar to that described in Jensen et al. (2014) (see Supplementary Methods 1 for detailed description). SMR was chosen from the average of the three (22°C) and two (30°C) lowest measurements of CO_2_ production rates as this is assumed to reflect measurements of inactive animals. After the experiment the spiders were re-weighed, placed in Eppendorf tubes and frozen for later analysis (lipid/protein content).

A second experiment was conducted, this time using group sizes of one, five, and twenty. Twenty-seven spiders (one group of 20 individuals, one group of five individuals, and two replicates of individual spiders) were obtained from each of nine spider colonies (Supplementary Table S1). The experiment was conducted using an almost identical approach as described above (for differences with the first round of experiments, see Supplementary Methods 1), however, both acclimation and the experiment were conducted only at 22°C.

The SMR was calculated as CO_2_ production in microliters per gram spider per hour (V_CO2_ μL/g/h) (Supplementary Methods 1). We used general mixed models (GMM) to analyse effects of group size, acclimation temperature and experimental temperature on SMR. The colony from which spiders originated was included as a random factor.

### Lipid/protein content

In parallel with the measurements of SMR, we assessed the effects of temperature acclimation and group size on lipid and protein content as an indirect measure of energy usage and starvation strategy. At the onset of the experiment, 25 spiders from each of 12 colonies were sampled and fed to satiation. The spiders were weighed to the nearest 0.01 mg with a Sartorius Laboratory Balance (type 1712; Göttingen, Germany) and divided between eight clear plastic cylindrical vials, 113 mm high and 240 mm in diameter, closed with a piece of foam rubber. Apart from one individual that was frozen at −20°C to establish baseline values of lipid/protein content at time zero, four spiders were housed individually and the remaining 20 spiders were placed in four groups of five. Two vials with individual spiders and two vials with groups of five spiders were placed into each of two temperature cabinets set to 22 and 30°C respectively, where they were kept without food. Of these vials, one per colony × group size treatment was removed from each temperature chamber after 13 days, and another after 26 days. These spiders were immediately frozen at −20°C until further analyses. At onset of the lipid:protein analyses, the wet weight of spiders was determined. Spiders were then dried in an oven for ~90 h at 60°C and dry weight determined. Percentage water content of spiders was calculated.

The dried spiders were placed in desiccators until lipid/protein content analysis was carried out (details in Supplementary Methods 2). As mentioned in the supplementary material, protein content was determined by measuring nitrogen content and then calculating protein content using a conversion factor (see Supplementary Methods 2). Using the fraction of lipid and protein, the individual energy content and the daily energy consumption rate were determined (Supplementary Methods 2).

GMM were used to calculate the effects of group size, experimental temperature and starvation duration (13 or 26 days) and their interaction on percentage water content (log transformed), percentage lipid content, percentage protein content, energy content (log transformed), and daily energy rate. The colony from which spiders originated was included as a random factor. Response variables were log transformed to meet model requirements when necessary.

### Feeding efficiency

The effect of group size on feeding efficiency was measured for individual spiders and groups of five spiders (Supplementary Table S1) at four different temperatures (18, 24, 30, 36°C). This experiment was repeated twice to account for the order in which different experimental treatments were conducted (Supplementary Methods 3 and Supplementary Table S2). We investigated whether initial spider and fly mass differed between individual and grouped spider treatments (Supplementary Methods 3).

To allow acclimation to group size, spiders were placed into petri dishes housing either a single or a group of five spiders some days before onset of the experiment (Supplementary Methods 3). On the day of the experiment, the petri dishes with spiders were placed into the incubators set to the experimental temperature 90 min before the onset of the experiment to allow temperature acclimation. Immediately before the experiment *Calliphora* flies were weighed to the nearest μg (start mass). At the start of the experiment, the petri dishes containing the spiders were removed from the incubator and one *Calliphora* fly per spider (one for the single spider treatment and five for groups of five spiders to secure same prey/spider ratio) was added, after which the petri dishes were returned to the incubator. The number of spiders feeding on a fly or flies in the petri dishes was scored immediately after the addition of the fly; each petri dish was scored every 10 min for 2 h from the first spider attack. Prior to the experiment, we verified that fly mass was not completely extracted after 2 h to ascertain that we accurately measured extraction efficiency. Petri dishes in which no spiders had attacked within 2 h from the start of the experiment were discarded.

Two hours from the time period of first attack in a petri dish, the petri dish was removed from the incubator and the spiders separated from the flies. The fly remains (including live flies) were weighed to estimate the total mass extracted over the feeding period (end mass).

We assessed the mass extracted by spiders from flies by subtracting the end fly mass from the start fly mass. This extracted mass was corrected for fly mass loss through dehydration during the experiment by subtracting with a correction factor (temperature dependent percentage) that was calculated for the different experimental temperatures (Supplementary Table S3).

Two measures of spider feeding efficiency were calculated. Because we found that larger spiders extracted more fly mass (Supplementary Results 2), we calculated the fly mass extracted per unit spider mass (*EpSM*) by dividing the extracted fly mass (*FM*) by the total spider mass in a petri dish (*SM*).

EpSM= FM/ SM

We observed that not all spiders fed during the entire duration of the experimental period; anything between zero and five spiders could be recorded feeding at a unit time per petri dish. For this reason, we calculated a second measure (*EpSMF*) that corrected for how long spiders fed by controlling for spider feeding time in addition to the mass of the spiders. This provided a measure of fly mass extracted per unit spider mass per unit of feeding time.

$$EpSMF=FM/\sum_{i}^{1}SC_{i}\;*\;SM/P$$

where *FM* is fly mass per petri dish, *SC*_*i*_ is the number of spiders feeding at observation time *i, SM* is the total spider mass in the petri dish and P is the number of spiders in the petri dish. Because this group feeding efficiency calculation includes cases where flies may have been fed upon by single spiders, it provides a conservative estimate of the benefits of group feeding.

GMM were run to assess the effects of group size and temperature on feeding efficiency. We also controlled for the number of starvation days (starvation duration) of the spiders in the analyses, as experiments at different temperatures were conducted across three days, meaning that spiders in the experiment conducted on the last day were starved for up to two days longer than spiders used in other experiments. The colony from which spiders originated and the experimental round (October or November) were included as random factors in the analyses. An interaction term between temperature and colony size was initially included in the analysis. This interaction was insignificant and was thus removed from the model. The significance of the final model was determined by a Chi-square test comparing this model to a null model which included starvation duration and the two random factors.

In addition to feeding efficiency, we also analyzed whether individual spiders or spiders in groups attacked prey faster, i.e., the propensity of single vs. groups of spiders to attack prey. The effects of group size and temperature on the time to the first attack were assessed using a GMM. The time to the first attack was log-transformed, and the effect of colony size and experimental temperature on the time of first attack assessed, while controlling for starvation duration. Spider colony and experimental round were included as random factors. We also tested for an interaction between colony size and temperature, but as it was insignificant, it was not included in the final analysis.

It could be argued that petri dishes with five spiders that were fed with five flies had a higher likelihood of spider-fly encounters than single spiders that were fed only one fly, and therefore that the time to attack is expected to be lower in petri dishes with more spiders and flies. Therefore, a resampling procedure was applied to assess whether the time to attack in spider colonies of five was significantly different to what would be expected from the attack incidences recorded for single spiders. In short, we artificially generated distributions of attack times for groups using the pool of single spider data and withholding the lowest attack time as the time to first attack from this group. The difference between the artificially generated distribution and the actual group values distribution was determined using a Kolmogorov-Smirnov test. We additionally determined if the difference between the artificial mean and true mean was significantly different from zero. For a detailed description, see Supplementary Methods 3.

All analyses were conducted in R v. 3.1.1 (R Development Core Team, 2014).

### Web building investment

To assess the temperature-dependent effects of group living on web-building investment, we randomly allocated spiders from each colony to groups of single spiders or five spiders over two experimental temperatures, 22 and 30°C (Supplementary Table S2). Spiders were weighed to the nearest 0.01 mg and were placed in clear plastic cylindrical vials (113 mm high; 24 mm diameter) with two attachment points for web building. Vials were placed in the respective climate cabinets without access to food or water. After three days the silk was collected from each vial. If molting had taken place, all remnants of exoskeletons were removed from the web. The web was weighed to the nearest 0.001 mg and web-building investment was calculated as the mass of web produced per unit mass of spiders. For analyses, silk mass was log-transformed and the effect of group size and acclimation temperature and their interaction on web production analyzed using a GMM, with colony identity included as a random effect.

### Desiccation resistance and water balance

We examined the effect of group size on water loss and survival rate under desiccation stress. One day before onset of the experiments spiders were fed to satiation with crickets. Spiders from each of the 14 colonies were randomly selected and placed in rectangular containers (11.5 × 11.5 × 6 cm) at different group sizes (Supplementary Table S2). From each colony (*N* = 14) there were four containers with one spider, one container with five spiders, one container with 10 spiders and one container with 20 spiders. Two sides of the containers were cut out and covered with gauze to ensure similar humidity levels in the container as in the surrounding chamber (see below). At the onset of the experiment four spiders from the same 14 colonies were frozen at −18°C for baseline measures of water content (see below).

Containers were weighed to the nearest 0.001 g with a Mettler Toledo balance before the spiders were introduced (type PJ360 Delta Range; Greifensee, Switzerland) such that total spider mass was measured from the total mass of containers and spiders. Containers with the spiders were placed in desiccation chambers and reweighed daily over a 44-day period to assess the desiccation-induced loss of mass. To correct for any mass loss related to the container itself 19 empty containers were used as control. All containers were randomly distributed into six hermetically closed tanks with glass lids. A layer of 2–3 cm silica gel on the bottom of the tanks ensured a constant low humidity (relative humidity [RH]) <5% (checked daily with iButton datalogger) and all tanks were placed in the climate room with constant temperature of 25°C. All containers with spiders were weighed daily in a room with a RH of around 25% and a room temperature of 25°C (~45 min per day). Any spiders that died during the duration of the experiment were weighed and frozen at −18°C for analysis of water content. After 44 days all spiders had died and the experiment ceased. The water content of all spiders was calculated by measuring spider mass before and after being dried in an oven at 60°C for 2 days.

To find the LT50-values (lethal time for 50% of the spiders), a dose-response curve with variable slope was fitted to the survival data and LT50-values were analyzed using the Graphpad Prism 6.0 program.

### Statistical analysis

All data, except where otherwise specified, were analyzed using JMP (version 10). For all general linear mixed models, backward selection was used to remove any insignificant interaction terms. The backward selection was based first on marginality, and secondly, if an analyses included several interaction terms with identical number of terms, on *p*-values. Main terms were not removed using backward selection. Only the results of the final models are presented.

## Results

### Standard metabolic rate

**Figure 1:**
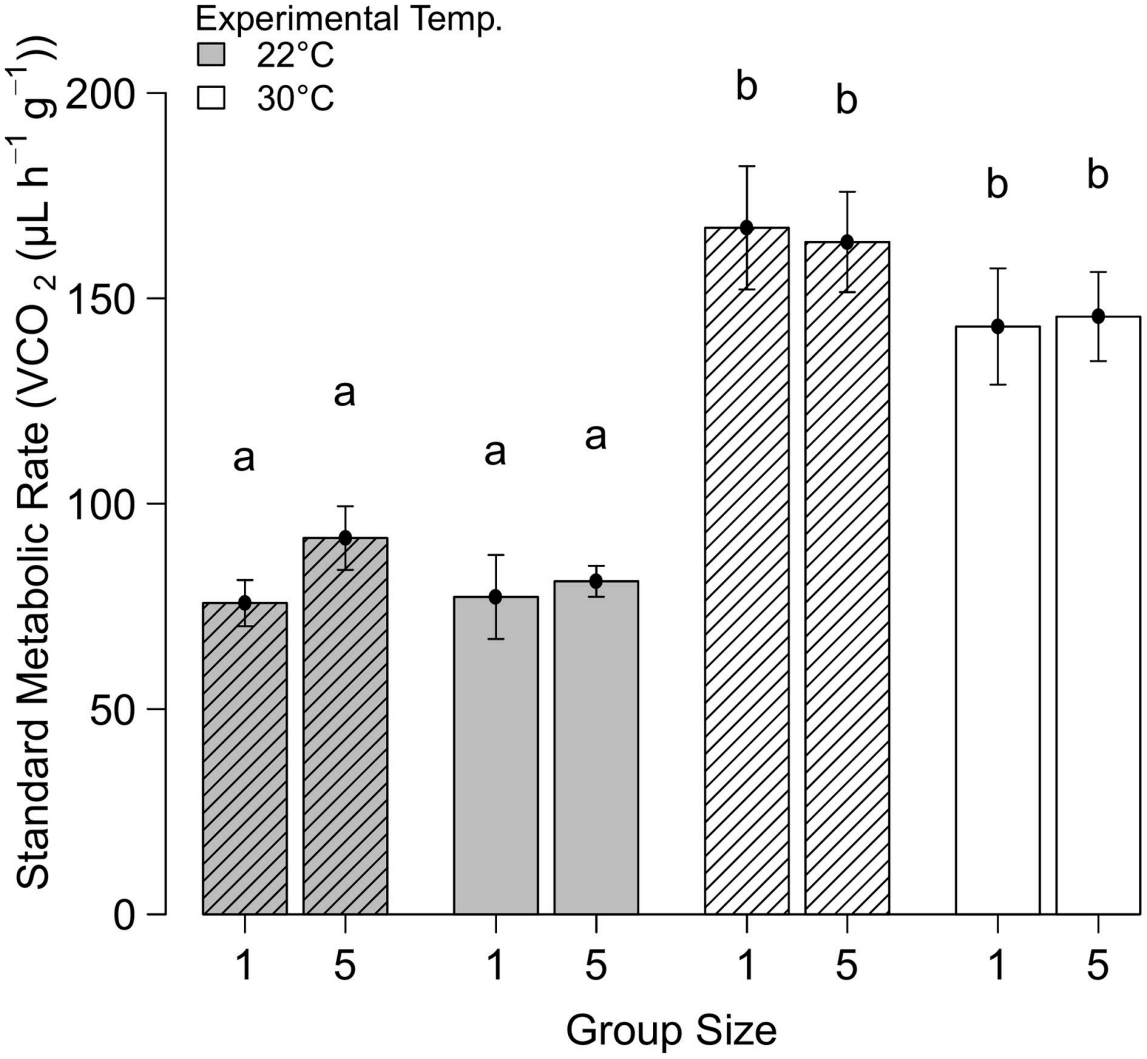
**The standard metabolic rate (mean ± SE) of *Stegodyphus dumicola* spiders kept individually and in group size of five, acclimated to two temperatures (22°C = clear and 30°C = hatched), and measured at 22 and 30°C**. Letters indicate significant differences.

**Figure 2:**
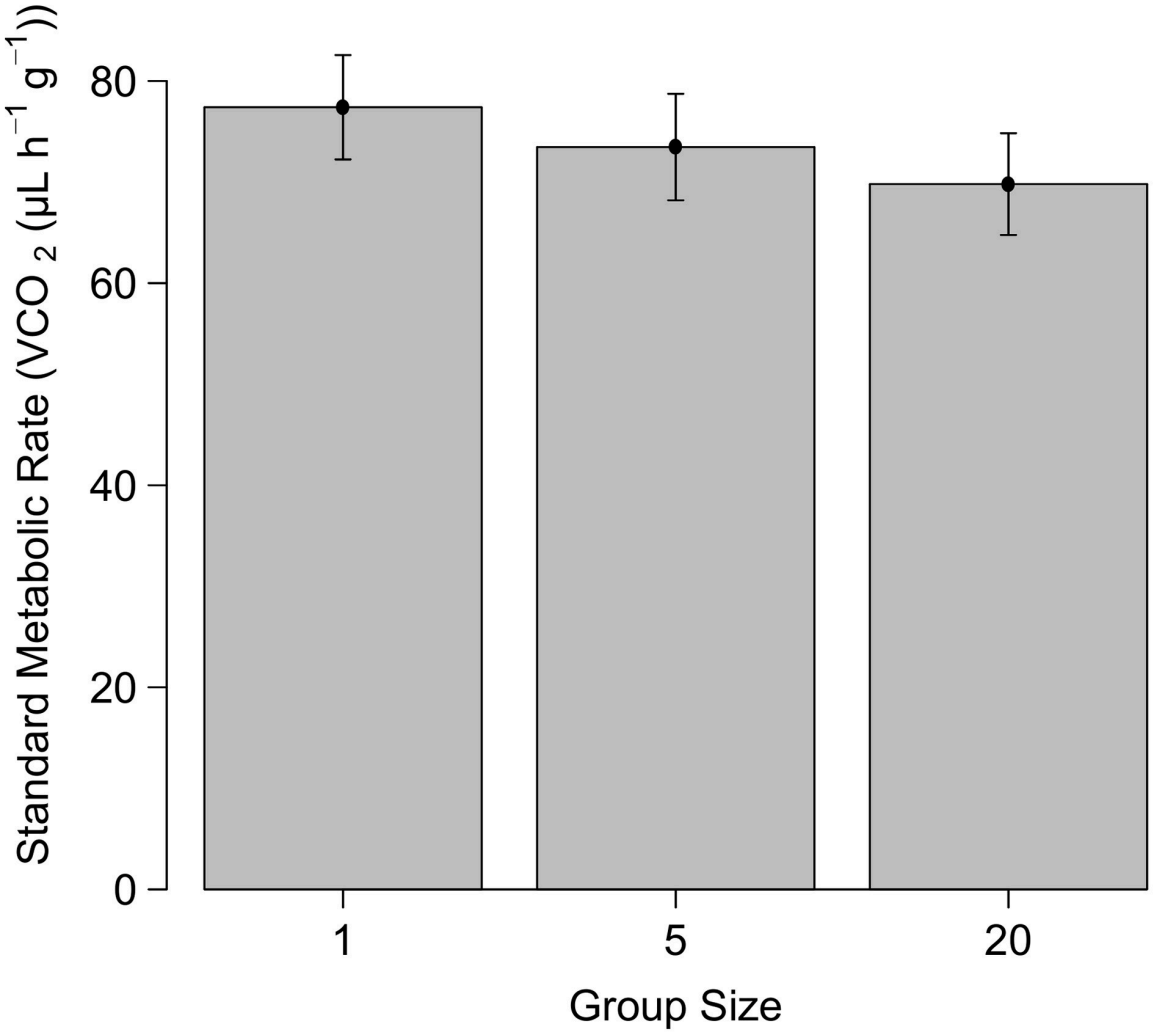
**The standard metabolic rate (mean ± SE) at 22°C of *Stegodyphus dumicola* spiders kept at different group sizes**.

The initial SMR experiment comparing individuals and groups of five did not reveal a significant effect of group size on SMR (*F* = 1.59; *P* = 0.431, *N* = 72), but the standard metabolic rate was much higher at 30°C than at 22°C (*F* = 1.49; *P* < 0.0001, *N* = 72, Figure 1). Acclimation temperature had no effect on SMR (*F* = 1.56; *P* = 0.055, *N* = 72). Similarly, in the second experiment (including a group size of 20) where SMR was only measured at 22°C, no significant effect of group size on the SMR was found (*F* = 2.38, *P* = 0.476, *N* = 48, Figure 2).

### Lipid/protein content

There was no significant effect of group size on percentage water content in starved spiders (*F* = 0.05, *P* = 0.8, DF = 1, DFDEN = 67.69, *N* = 82). Spiders lost more water when kept at 30°C than at 22°C (*F* = 8.56, *P* = 0.005, DF = 1, DFDEN = 67.82, *N* = 82). Similarly, spiders lost more weight at higher temperature (*F* = 1.69, *P* < 0.003, *N* = 84) and when starved longer (13 vs. 26 days, *F* = 2.81, *P* < 0.0001, *N* = 96).

We found no consistent effects of group size or temperature on the lipid, protein (converted nitrogen) or energy content (Supplementary Results 1; Supplementary Figures S1–S3) of *S. dumicola*. The three-way interaction between experimental duration, temperature and group size on energy content and lipid content, and the interaction between temperature and group size on protein content were significant (Supplementary Figures S1–S3 and Supplementary Tables S4–S6). Group size effects on energy content were only found in the 26-day treatment kept at 30°C, when groups of five spiders had a higher energy content than individual spiders (Supplementary Figure S1). For lipid content group size had an effect after 26 days: at 22°C, groups had a lower lipid content than individual spiders, while at 30°C the pattern was reversed (Supplementary Figure S2). No group size effects on protein content were found (Supplementary Figure S3).

### Feeding efficiency

**Figure 3:**
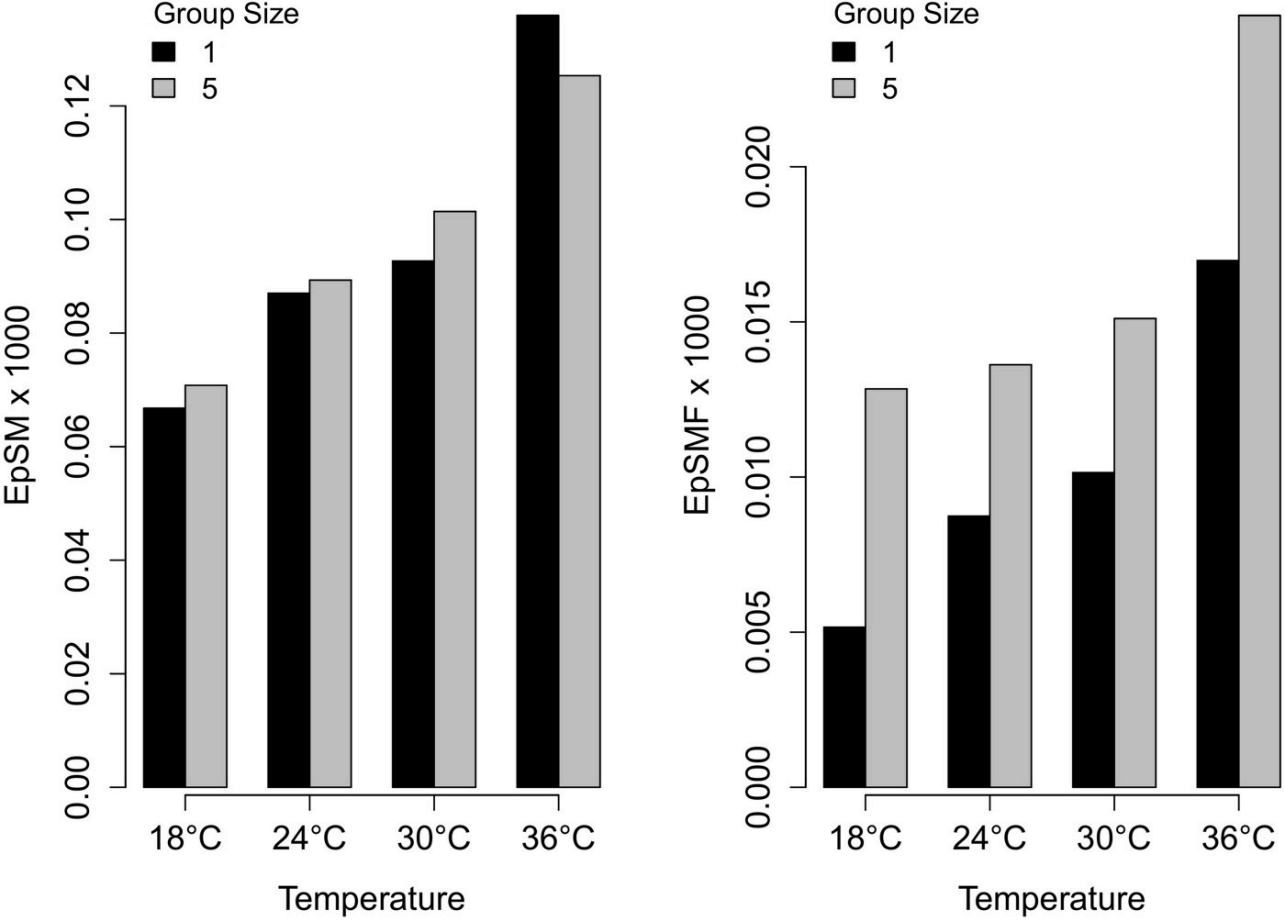
**The effect of temperature and group size on feeding efficiency of *Stegodyphus dumicola* spiders**. Left, fly mass extracted per unit spider mass over the duration of the experiment (*EpSM*); right, fly mass extracted per unit spider mass per unit feeding time (10 min) (*EpSMF*).

Both measures of feeding efficiency, namely fly mass extracted per unit spider mass, and fly mass extracted per unit spider mass per unit feeding time, increased with temperature (Figure 3; Table 1). The amount of fly mass extracted per unit mass of spiders did not significantly differ between group sizes (Figure 3, Table 1); whereas fly mass extracted per spider mass per unit time feeding was higher for groups than for single spiders (Figure 3, Table 1). This means that spiders in groups where multiple spiders were feeding on the same fly were more efficient, i.e., faster, in extracting mass from the fly.

**Table 1:** **Results from generalized mixed models examining the effect of temperature and group size on two measures of feeding efficiency in ***Stegodyphus dumicola***: fly mass extracted per unit spider mass over the duration of the experiment (***EpSM***) and fly mass extracted per unit spider mass per unit feeding time (10 min) (***EpSMF***)**.

	***EpSM***	***EpSMF***
*Whole Model p*	*1.4 × 10^−6^*	*2.8 × 10^−17^*
Intercept	–3.0^***^	–5.7^***^
Group size	6.8 × 10^−3 *NS*^	4.7 × 10^−1^^***^ (Group > Single)
Temperature	3.0 × 10^−2^^***^	4.5 × 10^−2^^***^
Starvation duration	–9.3 × 10^−2^^*^	–6.9 × 10^2 *NS*^

The whole model p-value is displayed in italics in the first row, and the estimates of the fixed predictor variables along with their p-values (asterisks) are displayed in the rows below (asterisks designate different significance levels

* *P ≤ 0.05*,

*** *P ≤ 0.001; NS = not significant)*.

Flies were more likely to be attacked (Supplementary Figure S4) and attacks occurred faster (Supplementary Figure S5) in groups of five spiders compared with single individuals. However, this is likely due to a higher encounter rate in petri dishes with five spiders and five flies as the resampling analysis revealed no significant difference between treatment groups (Supplementary Results 2).

### Web building investment

**Figure 4:**
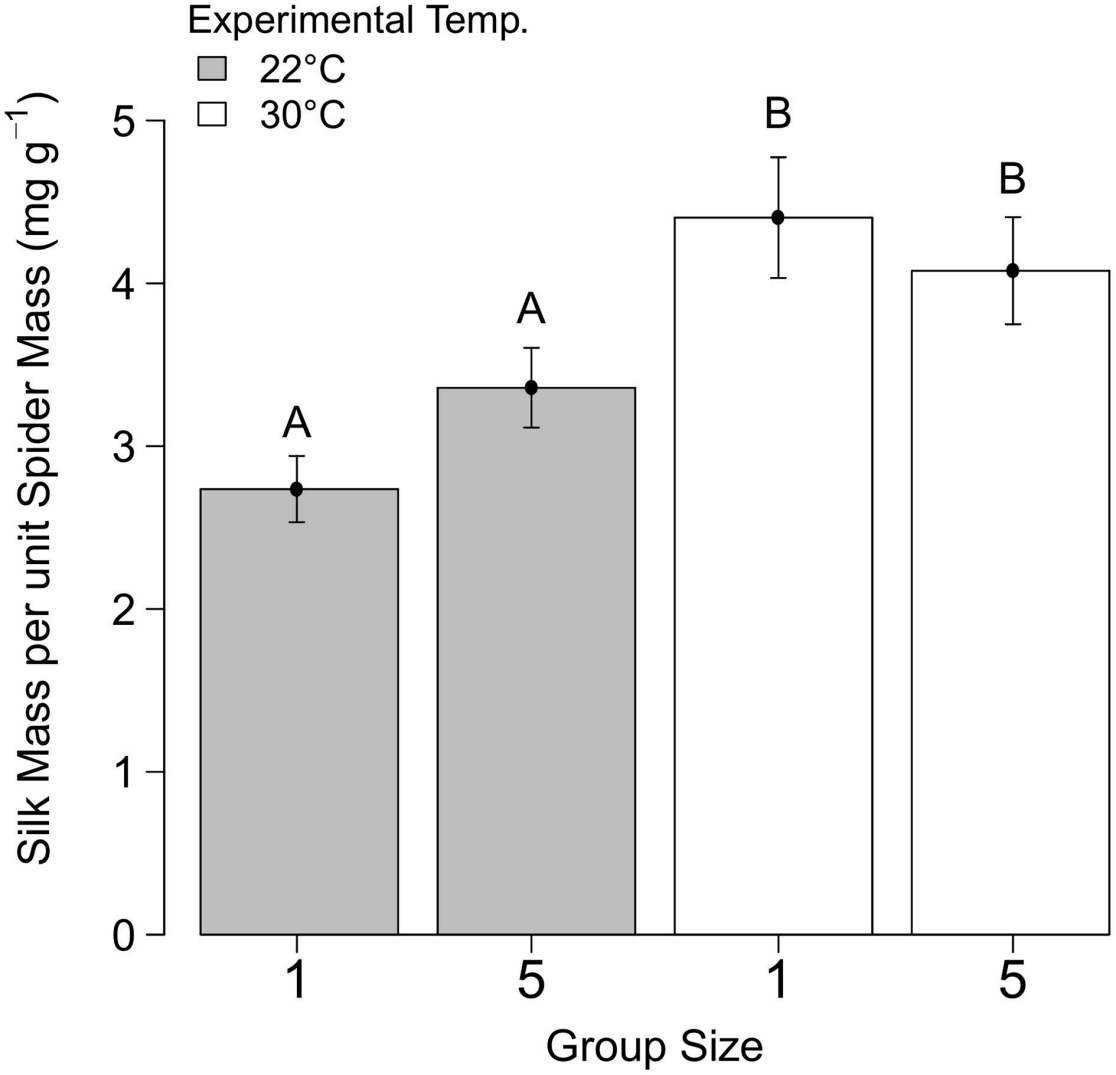
**The mean (±SE) silk deposition of *Stegodyphus dumicola* (mg) per spider (g) kept at two different group sizes (one and five) and two different temperatures (22 and 30°C) for 3 days**. Letters above bars indicate significant differences.

There was no effect of group size on web building investment (*F* = 1.19, DF = 1, DFDEN = 133.8, *P* = 0.28, *N* = 147). Spiders produced more silk at higher experimental temperatures (*F* = 21.93, DF = 1, DFDEN = 135.2, *P* < 0.0001, *N* = 147, Figure 4).

### Desiccation resistance

**Figure 5:**
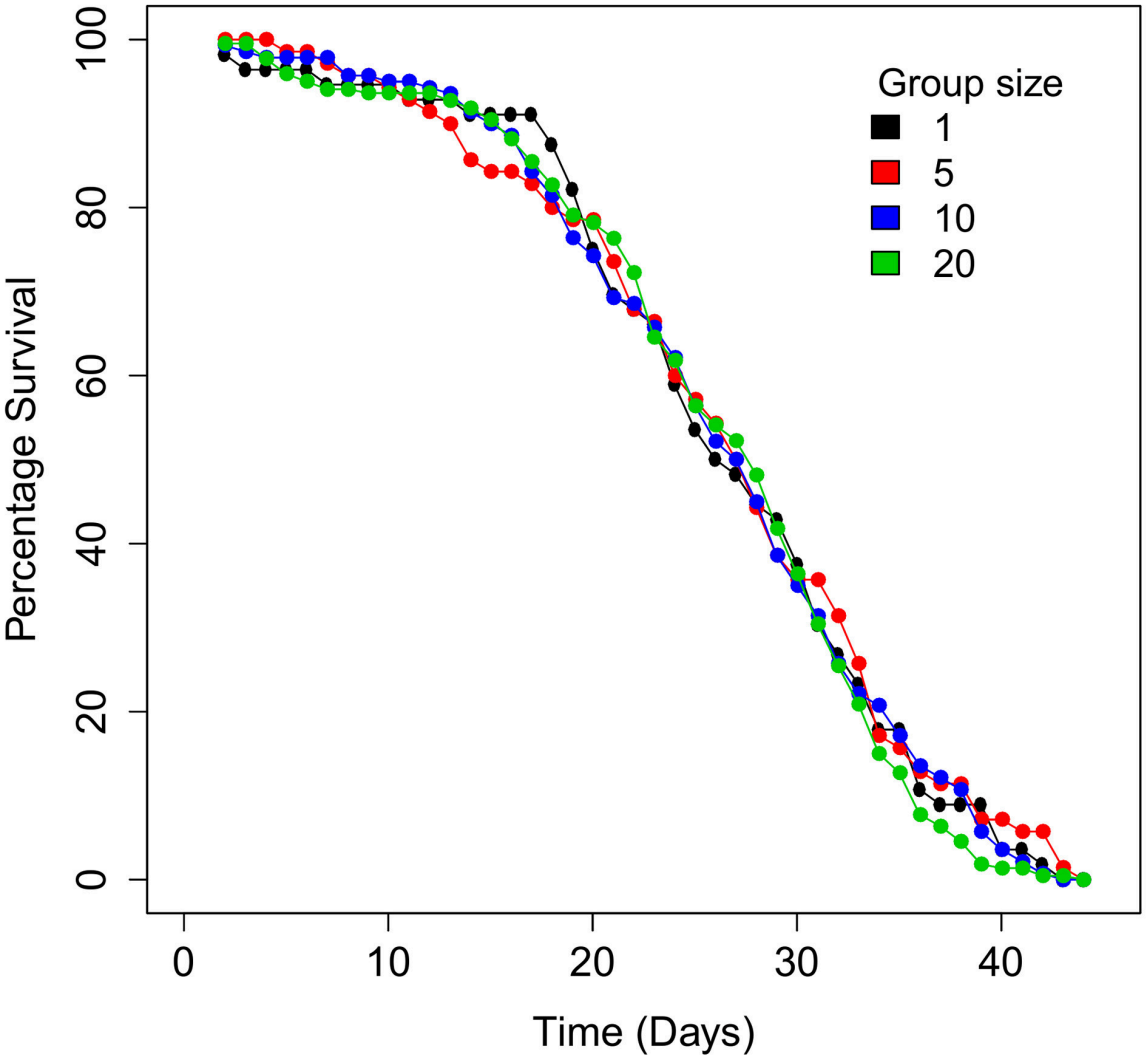
**The survival rate of *Stegodyphus dumicola* spiders at four different group sizes (1, 5, 10, and 20) subjected to desiccation in chambers with relative humidity of <5% over 44 days**.

**Figure 6:**
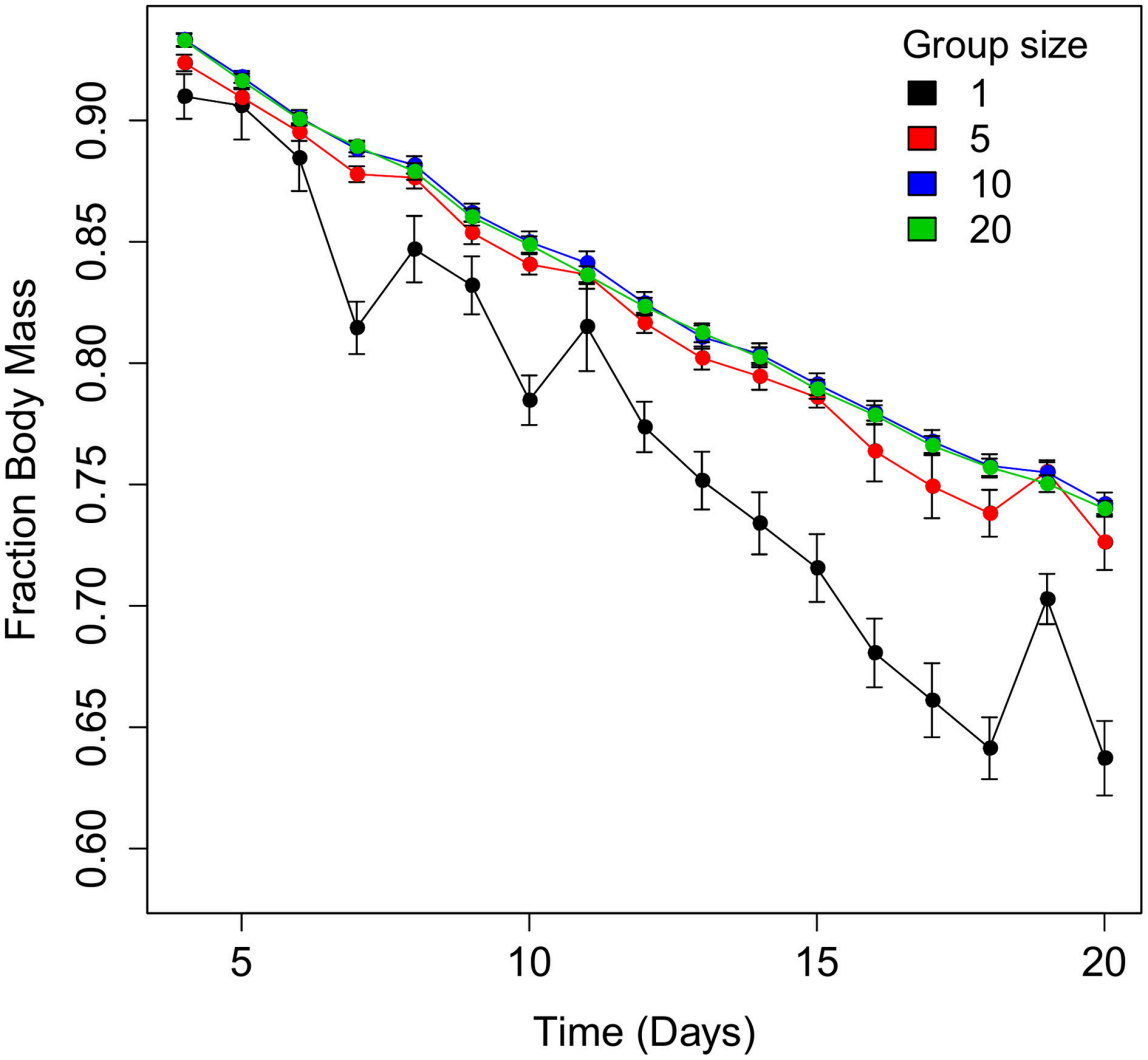
**The mean (±SE) mass loss rate between day 4 and 20 of *Stegodyphus dumicola* spiders subjected to desiccation at four different group sizes**. Spiders kept individually had a significantly higher mass loss rate than spiders kept in larger groups.

There was no effect of group size on the survival rate of spiders subjected to severe desiccation (Wilcoxon test, χ^2^ = 1.3647, DF = 3, *P* = 0.714, *N* = 556; Figure 5). Group size had a significant effect on mass loss rate (*F* = 3.1; *P* < 0.0001, *N* = 516, Table 2 and Figure 6) with solitary spiders losing mass faster than spiders in larger groups. Only mass loss data from day 4 to 20 were included in the statistical analysis due to a large drop in spider mass from day 1 to 3, and low survival after day 20. The faster mass loss of solitary spiders was, however, not associated with water loss, since water content at the end of the experiment was not affected by group size(*F* = 1.20; *P* = 0.310, DF = 410.7, *N* = 503, data on water content log transformed, Table 2).

**Table 2:** **The mean spider mass (±SE) at onset of experiment, the number of days (±SE) at which 50% of the spiders had died (LT50), the mean (±SE) mass loss rate of spiders over 17 days and mean (±SE) water content for the control spiders (measured at the onset of the experiment) and the four group sizes of spiders (1, 5, 10, and 20; measured at death of the spider)**.

**Colony size**	**# of colonies**	**Mean (±SE) initial mass per spider (mg)**	**LT50 (±SE)**	**Mean (±SE) mass loss rate(% day^−1^)**	**Mean (±SE) water content (mg water mg dw^−1^)**
Control	14	0.126 ± 0.02			1.99 ± 0.04
1	13	0.136 ± 0.02a	27.51 ± 1.02	−0.0162±0.0005	1.59 ± 0.05
5	14	0.128 ± 0.03ab	31.97 ± 1.05	−0.0123±0.0005	1.71 ± 0.04
10	14	0.126 ± 0.03ab	29.97 ± 1.02	−0.0119±0.0005	1.66 ± 0.03
20	12	0.117 ± 0.03b	29.14 ± 1.02	−0.0123±0.0005	1.63 ± 0.02

Values (mean ± SE) followed by a different letter within a column are significantly different from each other (P < 0.05).

## Discussion

We examined whether group living resulted in benefits in a suite of physiological and behavioral parameters related to bioenergetic effects in social spiders. We found positive effects of group size on two parameters, namely increased feeding efficiency and lowered mass loss during desiccation. Both feeding efficiency and mass loss during desiccation are relevant ecological factors as the former results in lowered predator exposure time, and the latter benefits social spiders that occupy arid, hot environments. On the other hand, we found inconsistent effects of group size on lipid and protein content, with group living only showing benefits at high temperatures, while for protein content effects were difficult to interpret as clear group benefits. While we observed the expected physiological response with higher SMR, higher web building investment and increased feeding efficiency at higher temperatures, we could not detect consistent interaction effects between temperature regime and group size on these parameters.

Feeding efficiency increased with temperature measures for single spiders and groups of five, and demonstrated that single and grouped spiders extracted equal amounts of prey per unit spider mass. However, groups of spiders extracted more prey than solitary spiders per unit feeding time, resulting in faster prey extraction during group feeding. This could result from a more efficient use of digestive enzymes, and can have the benefit of reduced time of exposure to predation. *S. dumicola* catches its prey in extensive capture webs that originate from the protective nest. Especially larger prey is usually consumed in the capture web, making the spiders prone to predation during feeding (Majer, 2015). Increased feeding efficiency through reduced feeding time may thus allow spiders to return to the safety of their nests more rapidly (Henschel, 1998). Spiders have extraoral digestion and rely on regurgitating digestive enzymes into the prey prior to nutrient extraction, and it is possible that quantitative effects of multiple feeding spiders result in synergic effects that increase prey extraction efficiency (Schneider and Bilde, 2008).

While feeding efficiency was higher in groups compared with individual spiders, we did not measure individual feeding efficiency in groups of spiders, although evidence suggests that intragroup competition exists. Other studies of *S. dumicola* have shown that spiders to arrive at a prey item first feed longer and gain more mass than second spiders (Amir et al., 2000), that contests over food and prey feeding sites (i.e., thorax vs. legs) occur (Whitehouse and Lubin, 1999), and that smaller spiders can be excluded by larger spiders at feeding sites (Whitehouse and Lubin, 1999). Levels of competition increase with feeding group size, hence reducing feeding efficiency (Whitehouse and Lubin, 1999). These results suggest that an optimal feeding group size may exists in social spiders. It has also been shown that larger groups of *S. dumicola* have improved survival, although their mean female body size is lower, which could be a result of feeding competition (Bilde et al., 2007). These data show complex cost benefit trade-offs that likely constrains optimal group size to intermediate size in *S. dumicola* (Bilde et al., 2007) and in the social spider *Anelosimus eximius* (Yip et al., 2008). Given the stationary nature of social spider nests, where groups are acutely depending on prey to arrive in their capture webs, cooperative foraging is one of the prerequisites for group living, and group living evolves in productive habitats (Majer et al., 2013, 2015; Majer, 2015). Higher efficiency of group feeding is thus likely an important adaptation in social spiders.

During a period of extreme desiccation (RH < 5%), we found that single spiders lost significantly more mass compared to spiders in groups, while we detected no difference among groups consisting of five, 10, or 20 individuals. Despite losing more mass, there was no difference in water content (mg water/mg dry mass) between solitary and grouped individuals at the time of death, and the difference in mass loss did not translate into lower survival of solitary individuals. This could indicate that the limiting factor in survival of *S. dumicola* during desiccation is water content, with spiders unable to survive if the water content dips below a certain threshold. This could explain why water content was similar for individual and grouped spiders. In much of its range, *S. dumicola* inhabits arid and hot environments in southern Africa, making it likely that water balance control is crucial for survival. This may be reflected in the long period in which spiders survived (up to 44 days). Clustering of individuals is an important behavioral adaptation for arthropods that are either susceptible to water loss due to high evaporation rates and/or that inhabit arid environments. Indeed, several studies reveal a lower weight loss rate (snails, Rojas et al., 2013; woodlice, Broly et al., 2014), water loss rate (dust mites, Glass et al., 1998; bed bugs, Benoit et al., 2007) and higher survival (Ivarsson and Jonsson, 2004) in larger groups compared to solitary individuals under desiccation stress. Aggregation in clusters reduces the body surface area exposed to the air (Allee, 1926; Broly et al., 2014) and can create a local microclimate of increased humidity for all individuals in the group (Schliebe, 1988). Both factors are likely to be important in social *S. dumicola* spiders: in the field, temperatures of up to 53°C have been recorded inside the nest and spiders tend to leave, and seek cooler shelter in the shade of the nest at particularly hot times, which may function to reduce body surface exposure to desiccation (Soydaner, 2013). In addition, during the desiccation experiment, spiders were found to cluster closely together in compact groups within their silken nest-structure, possibly to reduce desiccation stress. The thick silk nest and behavioral plasticity such as group size dependent huddling may interact with group size to retain relative humidity and reduce desiccation rates.

It is unlikely that single spiders lost more mass due to a higher silk use for web building as our study did not reveal higher investment of single spiders (and thus a higher dry mass loss). The measurements of CO_2_ production rate and protein loss revealed similar metabolic rates between group sizes, suggesting that differences in mass loss were not linked to elevated metabolic rate in single spiders. Finally, although we never observed attacking and killing of a conspecific by spiders in a group, we cannot rule out that feeding occurred on dead spiders in groups, acting to maintain body mass. In ants, groups of workers with larvae survived longer than groups without, presumably through feeding on the larvae (Modlmeier et al., 2013). In summary, while we found evidence for reduced body mass loss under desiccation in group living individuals, to link this result to group benefits, desiccation resistance should be supplemented with data on key performance parameters such as locomotor activity, prey capture success, and reproduction that are tightly linked to fitness. For example, body mass is tightly associated with fitness (Honek, 1993; Prenter et al., 1999; Kingsolver and Huey, 2008), if individuals in groups maintain a higher body mass than solitary individuals, this would translate into a reproductive benefit of group living.

We could not detect a significant effect of group size on SMR, implying that group living does not strongly affect SMR. Nevertheless, the relationship between SMR and group size has been difficult to predict in other arthropods. Groups of shield bugs (Tojo et al., 2005), termites (Muradian et al., 1999) and ants (Gallé, 1978) show a lower standard metabolic rate (SMR) compared to single animals. Other studies on ants (Brian, 1973; Lighton and Bartholomew, 1988; Lighton, 1989) and one study on caterpillars (Schoombie et al., 2013) indicated similar SMR-values to those found in this study, regardless of group sizes. Within the arachnids, two species of harvestmen exhibit an opposing response with one species showing a higher and the other a reduced SMR with increasing group size (Anderson, 1993). Also in ants, SMR appears to be influenced by social interactions as similar sized ant groups have a higher SMR in crowded circumstances (Cao and Dornhaus, 2008). It is perhaps unsurprising that SMR was not elevated during increased social interactions in *S. dumicola*, as they are sit-and-wait predators that remain mostly passive—a strategy that is linked to low metabolic rates (Anderson, 1970; Greenstone and Bennett, 1980). Comparing our results of SMR at 22°C (first experiment) against the relationship between body mass and SMR for spiders (Overgaard and Wang, 2012) revealed a much lower value (~50%) compared to other spiders. This could suggest that social spiders have a low SMR and that the group living strategy entails low energetic costs. However, further studies comparing social and subsocial (social juvenile phase, solitary adult phase) species in *Stegodyphus* and other genera are needed to verify this hypothesis.

There was no consistent effect of group size on the metabolism of lipid and proteins during starvation, in contrast to other studies that have found reduced lipid metabolization in larger groups (Santos et al., 2007). Lipid and energy content were the only factors that showed some evidence for the benefits of group living being dependent on environmental conditions (i.e., temperature), with group-living spiders having a higher energy and lipid content at high, but not low, temperatures. However, given the paucity of similar trends in the other experiments, we interpret these results with caution: it appears that environmental temperatures influence protein and lipid contents in a complicated manner. What remained unexplored in this study is whether the effects of group-living might be dependent on water stress, which is known to affect carbohydrate oxidation in arachnids (Kalra and Gefen, 2012).

Web building is a costly key aspect of spider ecology (Riechert, 1985; Tanaka, 1989) and is thus an important component of the energy balance of a spider. However, we found no advantage of group living for silk production. This is in contrast to findings that, in the field, web size does not increase proportionally with group size, suggesting that individuals in larger groups produce less silk per capita (Majer, 2015). The lack of group size effects on web investment could reflect the small group sizes used in the experiment, which are significantly lower than colony sizes found in the field (up to several hundred individuals). Nevertheless, larger groups of spiders have been shown to build larger nests that are better defended against predators and also act to regulate temperature (Seibt and Wickler, 1990; Henschel, 1998; Bilde et al., 2007; Soydaner, 2013; Unglaub et al., 2013). It is therefore likely that group benefits in the wild arise from the larger shared nest and capture web as well as reduced per capita investment in silk (Majer, 2015).

In this study, we investigated several bioenergetics variables relevant for group living in spiders in controlled lab settings that aimed to reflect some realistic environmental parameters experienced by *S. dumicola* in the wild. The spiders live in variable environments, which may undergo substantial changes between seasons, and sometimes during the course of a day. They are therefore accustomed to a wide range of environmental conditions. The temperature ranges and humidity conditions used in our experiments are similar to those experienced by spiders in the field (Soydaner, 2013). Furthermore, we selected suitable housing conditions for the web-building experiment that should not limit the behavior of the spiders. Although the spiders were confined to a container, available space was more than sufficient to allow for spiders to manipulate web building and web size. We observed that, at the end of the experiment, the vials were not completely covered in silk and unutilized space remained, even for groups of spiders. Additionally, due to the short duration of the web-building experiment, it is unlikely that the space available to the spiders would have affected the outcome of the experiment. Therefore, we are confident that the comparison of web investment between different group sizes is robust. However, our experimental group sizes were smaller than those most commonly found in the field—while some colonies have fewer than 10 individuals, others can house several hundred individuals. This could have affected the outcome of some of the experiments; for example, it is possible that SMR effects are only detectable at larger group sizes. Taken together, we cannot rule out that factors such as lab setting and experimental group size partly explain the non-significant results for some of the experiments presented.

In summary, we explored several traits that may confer bioenergetic benefits of group living in social spiders separately. Little evidence was found for temperature-dependent benefits of group living—and those we found appear to represent complex relationships that require further exploration. However, we found that spiders in groups fed more efficiently and lost less mass during desiccation compared with solitary individuals. Both of these benefits are likely to have important ecological implications as faster feeding reduces exposure time to predators, and these social spiders inhabit dry and arid environments with a high premium on water balance adaptations. Group living may serve to reduce body surface and hence desiccation, and the shared nest is expected to function in temperature and humidity regulation. Although we did not find group living advantages or costs in SMR, energy metabolism and web building, these variables should probably not be considered separately, instead cost/benefit analyses of group living should be incorporated into a wider framework of multiple variables that shape group size effects. Group living may confer other benefits such as predator defense and increased prey capture success (Nentwig, 1985; Rypstra and Tirey, 1991; Henschel, 1998; Guevara and Aviles, 2011). The bioenergetic variables that demonstrated a positive effect of group living (feeding efficiency and mass loss rate during desiccation) are likely to respond to a threshold group size above which these are not advantageous. It is most likely that we need to integrate the effects of multiple bioenergetic factors that shape the cost/benefit ratio of group living to improve our understanding of energetic benefits of group living in social spiders.

## Author contributions

BV, MG, AB, JB, JO, and TB designed and performed experiments. All authors contributed to the writing of the manuscript.

### Conflict of interest statement

The authors declare that the research was conducted in the absence of any commercial or financial relationships that could be construed as a potential conflict of interest.
