# Supplementary material for: Benefits of Group Living Include Increased Feeding Efficiency and Lower Mass Loss during Desiccation in the Social and Inbreeding Spider Stegodyphus dumicola
Source: Front Physiol. 2016 Feb 2;7:18. doi: 10.3389/fphys.2016.00018 (PMC4735397; doi:10.3389/fphys.2016.00018)
Supplement: Supplementary file 1 [file DataSheet1.docx]

**Supplementary Information**

**Spider rearing and experimental design**

**Table S1. Overview of the origin and number of *Stegodyphus dumicola* spider colonies used in each of the conducted experiments, the dates of their collection, their rearing conditions and developmental status at the time of the experiment, and the experimental procedure(s) each of the colonies was subjected to. Abbreviations: CC = kept in climate chamber, WW= wet weight, L:D = light:dark photoperiod.**

| Population | Coordinates | Number of colonies | Collection date | Rearing conditions | Developmental status | Experimental  procedure |
| --- | --- | --- | --- | --- | --- | --- |
| Weenen, Kwazulu-Natal | S° 28.8386,  E° 29.9816 | 12 | May 2012 | Room Temperature (22-23°C),  Natural photoperiod | 23 - 154 mg WW,  (sub)adult, egg sacs  absent | Metabolic rate 1, lipid protein content, web building |
| Pongola Nature Reserve, KwaZulu-Natal | S° 27.3333  E° 31.8667 | 15 | May 2012 | CC (25°C), 12-12 L:D | 16 – 860 mg WW, (sub)adult | Feeding efficiency 1 |
| Sodoma,  Limpopo Province | S° 23.5483,  E° 28.7303 | 17 | July 2013 | CC (25°C),  12-12 L:D | 4-18 mg WW,  juvenile | Metabolic rate 2 |
| Shingwedzi, Northern Kruger Park | S° 23.1167  E° 31.4333 | 15 | June 2012 | CC (25°C), 12-12 L:D | 33 – 688 mg WW,  (sub)adult | Feeding efficiency 2 |
| Colenso, Kwazulu-Natal | S° 28.7091,  E° 29.8091 | 14 | January 2013 | CC (25°C), 10-14 L:D | 50-322 mg WW,  adult, egg sacs present | Desiccation |

**Table S2. Overview of number of *Stegodyphus dumicola* colonies used per experiment, date of the experiment, duration of the experiment, the experimental temperature(s) at which each of the experiments was conducted, the acclimation temperature of the spiders (if relevant) and the different group sizes used for each of the five experimental setups. The standard metabolic rate experiment and the feeding rate experiment were repeated twice with different colonies.**

|  | Number of colonies | Experiment  Date | Experiment duration (days) | Experimental temperatures (°C) | Acclimation temperature (°C) | Group sizes |
| --- | --- | --- | --- | --- | --- | --- |
| Standard metabolic rate 1 | 9 | October 2012 | 18 | 22, 30 | 22, 30 | 1, 5 |
| Standard metabolic rate 2 | 9 | September 2013 | 18 | 22 | 22 | 1, 5, 20 |
| Lipid/protein content | 12 | October 2012 | 13, 26 | 22, 30 | / | 1, 5 |
| Feeding efficiency 1 | 15 | October 2012 | 8 | 18, 24, 30, 36 | / | 1, 5 |
| Feeding efficiency 2 | 15 | November 2012 | 8 | 18, 24, 30, 36 | / | 1, 5 |
| Web building  efficiency | 12 | October 2012 | 3 | 22, 30 | / | 1, 5 |
| Desiccation resistance | 14 | June-July  2013 | 44 | 25 | / | 1, 5, 10, 20 |

**Supplementary Methods**

1. **Standard Metabolic Rate (SMR)**

The rate of CO_2_ production (V_CO2_) of *S. dumicola* was measured using intermittent closed respirometry in an experimental setup that sequentially measures V_CO2_ in 16 metabolic chambers (12 metabolic glass chambers with spiders and four empty chambers). The experimental setup was similar to the one described in Jensen et al. ([2014](#_ENREF_1)). Briefly, two parallel 8-channels-multiplexers (RM Gas Flow Multiplexer, Sable Systems, Las Vegas, Nevada, USA) control the sequential flushing and closing of the metabolic chambers such that the stop-flow respirometry system was allowed to obtain repeated measures of V_CO2_ in 16 parallel metabolic chambers over a 21-hour period. During the flush phase the metabolic chambers were perfused with CO_2_ stripped air (air passing through a soda lime column (MERCK Millipore, Darmstadt, Germany) at a fixed rate of 200 ml min^-1^. Airflow was controlled by an adjustable mass flow meter (Side-Trak, Sierra Instruments, Monterey, California, USA) controlled by a flow controller (MFC 2-channel v. 1.0, Sable Systems, Las Vegas, Nevada, USA). After the flush phase the metabolic chamber was closed while the remaining 15 chambers were flushed sequentially in a similar manner such that the duration of the closed phase was 15 times the duration of the flush phase. The air leaving the metabolic chambers passed a calcium chloride column (AppliChem, Darmstadt, Germany) to remove water before entering a CO_2_ analyzer (Li-6251 CO2 Analyzer, LI-COR Environmental, Lincoln, Nebraska, USA). To optimize the signal-to-noise ratio the opening time was set at five minutes at 22°C and three minutes at 30°C (giving a closing time for 22°C at 15 x five minutes and for 30°C 15 x three minutes). Using these flush times, 15 and 26 independent measurements of V_CO2_ were obtained at 22°C and 30°C respectively. The first five measurements were always discarded due to the possible confounding effects of handling. The standard metabolic rate was then estimated using the average of the three lowest remaining measurements during the experimental period for 22°C, and the two lowest measurements for 30°C.

A similar approach was used for the second experiment; however, the opening time was set to 7.5 minutes (closing time for each chamber was 15x7.5 minutes) and ten independent measurements were obtained of V_CO2_ at 22°C.

*Analysis of metabolic data*

The raw data of fractional CO2 content from the air flushing the metabolic chambers was processed by a script in Mathematica (version 7.0, Wolfram Research, Champaign, Illinois, USA), which automatically identified the start, found the baseline value of each CO2 top and integrated the area between the graph and baseline. The signal from all measurements was examined individually and all recordings that were abnormal discarded (typically if some measurements were beyond the limits of the CO_2_ analyser). All measurements were corrected by subtracting the average value of CO2 production found in the empty chambers (resulting from a minor CO2 diffusion into the system). The CO2 production output from Mathematica was transformed into microliters per gram spider per hour (VCO2 μL/g/h) and the standard metabolic rate (SMR) was estimated using the average of the two lowest measures of CO2 at 22°C and the three lowest measures at 30°C for every day of measurements at the given temperature.

1. **Lipid protein content**

*Determination of lipid content*

The analysis of lipid content was carried out using a Soxhlet apparatus. A round-bottomed boiling flask containing petroleum ether (bp 40-60°C) was heated in an oil bath and connected to an extraction chamber with a bypass sidearm conducting solvent vapour and a siphon arm refluxing the organic solvent. A condenser with water as coolant was placed on top of it. Each sample was placed in a small tin container and transferred to the extraction chamber (20 containers at a time) and refluxed for approximately 48 hours (> 40 rounds of reflux). Afterwards the samples were left for one day in the fume hood to allow most of the remaining petroleum ether to vaporise, before samples were placed in the oven at 60°C to dry for three to four days. After the drying period the samples were weighed to the nearest 0.01 mg with a Sartorius Laboratory Balance (type 1712; Göttingen, Germany) and lipid content was calculated from the difference in mass before and after removal of the total lipid store.

*Determination of protein content*

The material remaining from the lipid extraction (above) was used for protein analysis (CN analysis). The dried samples were pulverized with metal balls (50 Hz in 2 min) using a TissueLyzer LT (Qiagen, Copenhagen, Denmark). Subsequently ̴2 mg of the tissue powder was weighed to the nearest 0,001 mg (Mettler Toledo AX26; Greifensee, Switzerland) and packed in tinfoil. The samples were combusted using He in NA 2000 N-Protein (Fisons Instruments, Italy) and the N content was calculated relative to standards with known C:N ratios (Cysteine, Atropina, and Sulfanilamid). Protein content was calculated using a protein:nitrogen factor of 6.25 (AOAC 2000).

The percentage water content of spiders was calculated as the difference between the wet and the dry mass, divided by the wet mass. Percentage lipid content and percentage protein content were calculated as the lipid and the protein content divided by spider dry mass respectively. Spider energy content (kcal) was calculated as (wet body mass (g)*fraction of dry mass* fraction of lipid*9.4 kcal/g) + (wet body mass (g)*fraction of dry mass* fraction of protein*4.25 kcal/g). A daily energy consumption rate (cal/g/day) was then calculated from the difference between the initial and final energy content of the spider assuming that each spider had the same energy density as controls.

1. **Feeding efficiency**

Experimental setup

**Day 1**: Four groups of six females were randomly selected from each of 15 colonies and placed into cylindrical vials (9 cm long, 3.5 cm in diameter). All females were of approximately equal size. Construction of a catching web inside the vials was allowed for 24 hours. **Day 2**: Six *Calliphora* flies were added to each vial to ensure that spider groups were fed equally prior to onset of the experiment. The spiders were allowed to capture and feed on flies for 24 hours. **Day 3**: Spiders were removed from each of the vials and separated into two different group sizes: a single spider, and groups of five. Spiders were weighed to the nearest mg so that feeding efficiency per mass unit of spider could be calculated. In groups of five spiders, all five spiders were weighed together and their weight averaged. Spiders were placed into petri dishes (5 cm diameter) and allowed to construct a catching web.

**Day 6, 7 and 8**: Experiments were conducted at four different temperatures (18°C, 24°C, 30°C, 36°C), which represents the range of temperatures at which spiders feed (unpublished data). Because different temperature treatments could not be conducted simultaneously, experiments were conducted over three days, with all experiments for one experimental temperature being conducted concurrently. In the first set of experiments the temperature order was: day 6: 24°C and 30°C , day 7: 18°C and day 8: 36°C. During the second set of experiments a reversed order was used (i.e. 18°C and 36°C on day 6, 24°C on day 7 and 30°C on day 8).

We used an ANOVA analyses (with log transformation of spider and fly mass data to meet model assumptions) to test whether initial spider masses and fly masses differed between petri dishes with individuals or groups of spiders.

We tested if larger spiders extract more fly mass using a generalized linear model (normal distribution with identity link) to assess the effect of log-transformed spider mass (controlling for experimental temperature) on fly mass extracted. For these analyses, we only used data on successful attacks of single spiders. This provided individual fly mass and individual spider mass data. A significant relationship between spider mass and extraction would indicate that feeding efficiency should not be assessed only in terms of the colony size of spiders, but also in terms of the mass of the spiders in the colonies.

Fly dehydration correction factor.

To account for fly mass lost through dehydration during the course of the experiment, the end mass of all unconsumed flies at 120 minutes was subtracted from their mass before onset of the experiment. These calculations were only conducted for petri dishes with a single spider and fly, as in most petri dishes with five spiders at least some of the flies were attacked. The percent fly mass lost due to dehydration was then calculated for each experimental temperature (see Table S3).

**Table S3. Percent *Calliphora* fly mass lost due to dehydration during a two-hour exposure to four different experimental temperatures. Standard errors (SE) and the sample size on which these calculations were based are also indicated.**

| Experimental temperature (°C) | Percent mass loss ± SE | Sample size |
| --- | --- | --- |
| 18 | 6.7 ± 2.4 | 14 |
| 24 | 10.4 ± 7.9 | 14 |
| 30 | 11.5 ± 5.9 | 9 |
| 36 | 14.3 ± 7.6 | 10 |

To verify if the rate of mass loss of flies due to dehydration was constant, we assessed fly dehydration at 30-minute intervals at two of the test temperatures: 24°C and 36°C in November 2014. Thirty-five flies were weighed, put in Petri dishes and placed in an incubator set to the test temperature. Every thirty minutes five flies were removed from the incubator and their post-dehydration mass determined. Percentage mass lost to dehydration was then calculated and plotted against time. Visual inspection indicated that linear relationships explained the rate of mass loss better than quadratic functions in both test temperatures. At 24°C mass loss due to dehydration could be predicted by 1.58 + 1.88*Time (R^2^ = 0.87), while at 36°C mass loss due to dehydration could be predicted by -1.1 + 5.81*Time (R^2^ = 0.97). Because we found that the dehydration rate did not change over time, the experiment was not repeated at different temperatures.

Resampling procedure

A difference in attack time could result from a higher opportunity for random prey-predator encounters in petri dishes with five spiders and five flies, rather than a pure group-size effect. To test this, we used a resampling procedure, 119 (= the same number of groups of five spiders as in our experiment) “random” groups of five spiders each were created by sampling the time to first attack (including incidences of where spiders did not attack) from the pool of times to first attack recorded for single spiders. Because experimental temperature and starvation duration did not predict time to first attack, resampling was conducted from single spiders tested at all experimental temperatures and starvation durations. Within a randomly generated group of five times to first attack, sampling was conducted from the pool of single spider times to first attack data without replacement. The minimum time to attack amongst the randomly selected times was taken to be the time to first attack for each randomly generated group. For these 119 random colonies the distribution of time to first attack were compared to those of the real 119 colonies using a Kolmogorov-Smirnov (K-S) test. In addition, the mean time to first attack of the 119 random colonies was computed and subtracted from the true mean time to first attack. The above procedure of computing 119 random measures of time to first attack was repeated 10 000 times, and the number of instances that the K-S test was significant and the number of instances that difference between the random and the true mean time to first attack was larger or smaller than zero computed. The assumption was made that, if the K-S test was significant in more than 95% of iterations, or if the difference between the random and the true mean time to first attack was larger or smaller than zero in more than 95% of iterations, the true time to first attack was significantly different to expected from random.

**Supplementary Results**

1. **Lipid/protein content.**

**
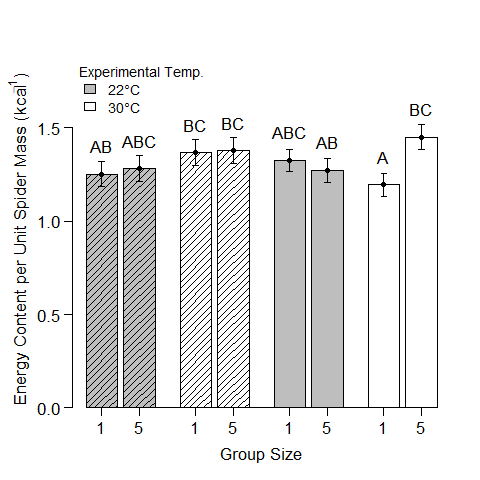
**

**Supplementary Figure S1. Mass-corrected energy content of *Stegodyphus dumicola* spiders kept at two group sizes (1 or 5) at two different temperatures (22°C and 30°C). Energy content was measured after 13 days (hatched) and after 26 days (clear). Letters above the bars represent significant differences.**

**Table S4. Overview of the fixed effects of generalized mixed models investigating the effect of group size (GroupSize: 1 or 5), experimental temperature (Temp: 22°C or 30°C) and experimental duration (Day: 13 or 26 days) on the energy content of *Stegodyphus dumicola* spiders. Significant effects are indicated in bold**.

| Effect | DF | F-value | P-value |
| --- | --- | --- | --- |
| Day | 66.72 | 0.05 | 0.8 |
| GroupSize | 62.41 | 2.45 | 0.12 |
| Temp | 62.53 | 2.77 | 0.10 |
| Day*GroupSize | 62.41 | 1.13 | 0.29 |
| Day*Temp | 62.53 | 1.13 | 0.29 |
| GroupSize *Temp | 62.62 | 3.50 | 0.066 |
| **Day* GroupSize *Temp** | **62.62** | **4.49** | **0.038** |


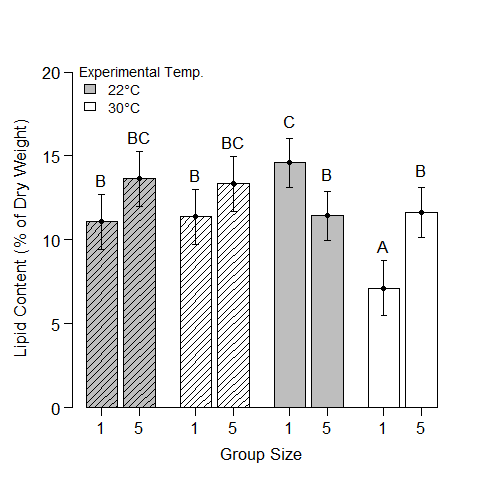


**Supplementary Figure S2. Percentage lipid content of *Stegodyphus dumicola* spiders kept in two group sizes (1 or 5) at two different temperatures (22°C or 30°C). Energy content was measured after 13 days (hatched) and after 26 days (clear). Letters above the bars represent significant differences.**

**Table S5. Overview of the fixed effects using generalized mixed models investigating the effect of group size (GroupSize, 1 and 5), experimental temperature (Temp, 22 and 30°C) and experimental duration (Day, 13 and 26 days) on the lipid content of *Stegodyphus dumicola* spiders. Significant effects are indicated in bold.**

|  | DF | F-value | P-value |
| --- | --- | --- | --- |
| Day | 67.69 | 1.45 | 0.23 |
| GroupSize | 63.88 | 2.54 | 0.12 |
| Temp | 63.96 | 3.89 | 0.053 |
| Day*GroupSize | 63.88 | 0.76 | 0.39 |
| Day*Temp | 63.96 | 3.89 | 0.053 |
| GroupSize*Temp | 63.96 | 3.69 | 0.06 |
| **Day*GroupSize*Temp** | **63.96** | **5.0** | **0.03** |


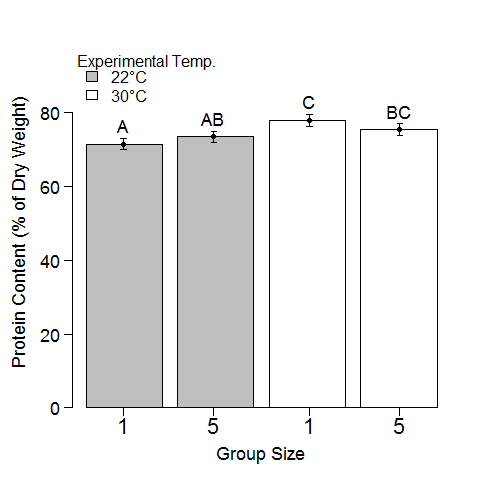


**Supplementary Figure S3. Percentage protein content (based on nitrogen conversion) of *Stegodyphus dumicola* spiders kept in two group sizes (1 or 5) at two different temperatures (22°C or 30°C). Duration of the experiment (13 or 26 days) had no effect on the percentage protein content. Letters above the bars represent significant differences.**

**Table S6. Overview of the fixed effects using generalized mixed models investigating the effect of group size (GroupSize, 1 and 5), experimental temperature (temp, 22 and 30°C) and experimental duration (Day, 13 and 26) on the protein content (based on nitrogen conversion) of *Stegodyphus dumicola* spiders. Significant effects are indicated in bold.**

|  | DF | F-value | P-value |
| --- | --- | --- | --- |
| Day | 70.9 | 0.98 | 0.33 |
| GroupSize | 66.93 | 0.04 | 0.8 |
| **Temp** | **67.04** | **15.2** | **0.0002** |
| **GroupSize*Temp** | **67.04** | **4.38** | **0.04** |

1. **Feeding efficiency**

Effect of spider mass on extraction.

Although spiders were randomly assigned to petri dishes containing single or groups of five spiders, single spiders were significantly larger than the mean mass of spiders in petri dishes containing a colony of five spiders (F = 5.06, df = 166, p = 0.026, R^2^ = 0.030). Mean initial mass of flies did not significantly differ between petri dishes containing single spiders and colonies of five spiders (F = 0.45, df = 166, p = 0.515, R^2^ = 0.003). General linear models indicated that larger spiders extract more fly weight when controlling for temperature effects (F = 17.33, df = 61, model p < 0.001, R^2^ = 0.362). P-values for spider weight and temperature were both < 0.001. Therefore we corrected for the effects of spider mass on feeding efficiency (see results).

Time to first attack and resampling procedure

In petri dishes where flies were added to colonies of five spiders, at least some flies were attacked in almost all instances, while flies added to single spiders were attacked in 60% of cases (Supplementary Figures S4 and S5). Most attacks on flies happened within the first 20 minutes of the experiment. Results from a GMM model indicated that the time to the first attack of spiders on flies was earlier in colonies of five spiders than in single spiders (p = 3.74 x 10^-4^, Supplementary Figure S5), while experimental temperature (p = 0.79) and starvation duration (p = 0.65) had no significant effect on time to first attack (whole model p = 2.04 x 10^-7^).


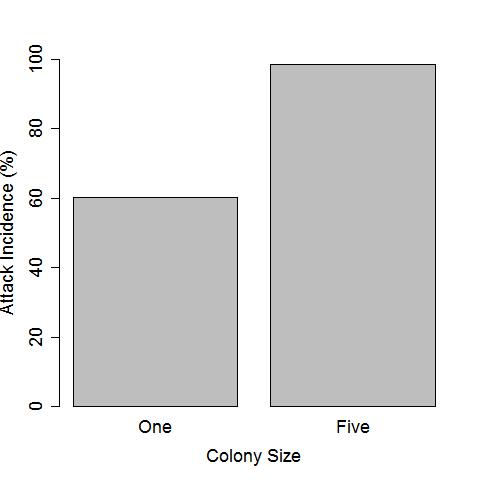


**Supplementary Figure S4. The likelihood with which single and colonies of five spiders attacked flies. Results have been summarized across experimental temperatures.**


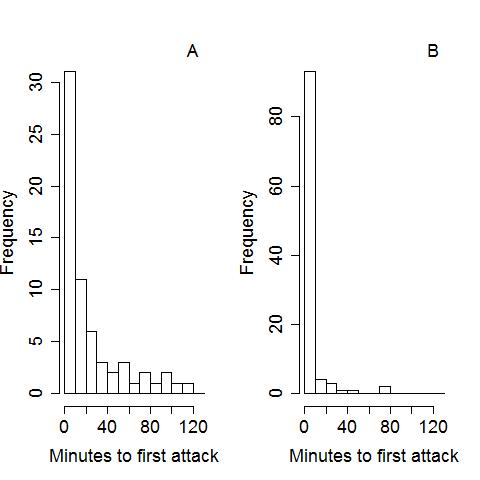


**Supplementary Figure S5. Histogram of the timing of first spider attack on flies. Spiders were kept individually (A) and in colonies of five (B). Note the different y-axes on the two figures.**

The distribution of the true time to first attack in groups of five spiders was only significantly different to that of randomly generated colonies in two out of 10 000 instances, indicating that in almost all instances spiders in groups did not attack faster than would be expected given the time to first attack observed in petri dishes with single spiders. The difference between the mean time of first attack in the 119 randomly generated and 119 true colonies was negative in 9054 iterations (i.e. observed data showed that spiders attacked later than expected from random), zero in 34 iterations (i.e. no difference between true results and expectation) and positive in 912 iterations (i.e. observed data showed that spiders attacked earlier than expected from random). Therefore, although the time to first attack was shorter in randomly generated colonies than in true colonies 90.5% of the time, this was not a significant effect, as α was considered to be 0.05.

**References**

Jensen, P., Overgaard, J., Loeschcke, V., Schou, M.F., Malte, H., and Kristensen, T.N. (2014). Inbreeding effects on standard metabolic rate investigated at cold, benign and hot temperatures in *Drosophila melanogaster*. *Journal of Insect Physiology* 62**,** 11-20. doi: 10.1016/j.jinsphys.2014.01.003.
